# Minimally invasive far lateral debridement combined with posterior instrumentation for thoracic and lumbar tuberculosis without severe kyphosis

**DOI:** 10.1186/s13018-020-01703-9

**Published:** 2020-06-16

**Authors:** Wei Xiong, Bing Yu, Yao Zhang, Chunxiao Wang, Xiaojie Tang, Haifei Cao, Xibing Zhang, Qinyong Song, Fang Tan, Jiangwei Tan

**Affiliations:** Department of Spine Surgery, Yantai Affiliated Hospital of Binzhou Medical University, No. 717, Jinbu Street, Yantai, 264000 Shandong China

**Keywords:** Minimal invasive surgery, Pedicle screw fixation, Spinal tuberculosis, Far lateral approach

## Abstract

**Background:**

Anti-tuberculous therapy (ATT) alone cannot easily cure spine tuberculosis (STB) though it is the most essential treatment. Many studies have confirmed the efficacy of the surgical treatment of STB through anterior, anterolateral, posterior debridement, and intervertebral fusion or combined with internal fixation. However, the conventional surgical approach requires extensive exposure of the affected areas with high rates of morbidity and mortality. Recently, minimally invasive surgery has come into use to reduce iatrogenic trauma and relevant complications. Here, we introduced a novel technique for the treatment of thoracic and lumbar spine tuberculosis: minimally invasive far lateral debridement and posterior instrumentation (MI-FLDPI). In this study, we evaluated the technical feasibility, the clinical outcomes, and the postoperative complications.

**Methods:**

We did a prospective, non-randomized study on this new technique. Twenty three patients (13 males) with thoracic or lumbar spine tuberculosis who underwent minimally invasive far lateral debridement and posterior instrumentation were included in the study. The preoperative comorbidities, operation duration, intra-operative hemorrhage, Cobb’s angles, and postoperative complications were recorded and analyzed. Clinical outcomes were evaluated by Visual Analog Scale (VAS), Oswestry Disability Index (ODI), neurological recovery, and eradication of tuberculosis. Radiological outcomes were evaluated by changes in Cobb’s angle and fusion status of the affected segments.

**Results:**

The patients were followed for an average of 19 months (ranging from 12 to 36 months). At the final follow-up, CRP and ESR of all patients were normal. The VAS and ODI were significantly improved compared with preoperative values (*P* < 0.05). No evident progression of the kyphotic deformity was found after surgery. Twenty two patients showed spontaneous peripheral interbody fusion 1 year after surgery. There were no failure of the instrumentation even though a young female with drug-resistant tuberculosis showed no sign of interbody fusion at the third year follow-up. All the patients with preoperative neurological deficit showed complete recovery at the final follow-up.

**Conclusions:**

MI-FLDPI using expandable tubular retractor could be recommended to treat thoracic and lumbar spine tuberculosis for the advantages of less trauma, earlier recovery, and less complications. Spontaneous peripheral interbody fusion was observed in nearly all the cases even without bone grafting.

## Introduction

Spinal tuberculosis (STB), which most commonly affects the thoracic and thoracolumbar spine [1, 2], accounts for nearly half of the skeletal tuberculosis [3]. Drugs alone cannot easily cure STB, although it is the most essential treatment. Resistance to first-line anti-tuberculous drugs have been observed in 8–12% of the tuberculosis patients [4]. Neurological deficit and deformity are the worst complications of STB. In patients who were treated conservatively, the natural tendency was healing with progressive anterior collapse until bony fusion occurs, often leaving 60° or more of residual kyphosis [5]. Early prevention and deformity correction along with anti-tuberculous therapy (ATT) are the fundamentals [6, 7].

Surgical treatment for STB includes anterior or posterior debridement, instrumentation, and fusion [8, 9]. Moon [10] found posterior instrumental stabilization and anterior interbody fusion that were helpful in arresting the disease early, providing early fusion, correcting the kyphosis, and preventing progression of the deformity. However, the comparatively large surgical trauma was likely to lead to more complications [11]. In recent years, with the development of new techniques such as direct or extreme lateral interbody fusion (DLIF, XLIF), endoscopic surgery and percutaneous pedicle screws, minimally invasive surgery has come into use for the treatment of STB [12–14].

In this study, we introduced a novel technique for the treatment of thoracic and lumbar spine tuberculosis: minimally invasive far lateral debridement and posterior instrumentation (MI-FLDPI). To our knowledge, this technical approach has not been described in available literatures. We evaluated the technical feasibility, the clinical outcomes, and the postoperative complications. The advantages of this technique were discussed by comparing with other minimally invasive procedures.

## Methods

This is a prospective clinical outcome study, which was approved by the ethics committee of our institution. Twenty three patients diagnosed with thoracic or lumbar spinal tuberculosis who underwent MI-FLDPI performed by the senior author from January 2013 to September 2018 were enrolled in this study. There were 13 males and 10 females aged from 17 to 71 years old with an average of 51 years. The demographic and clinical information about the patients was shown in Table 1.

**Table 1:** Demographic and clinical information of the patients

	Number
Total patient number	23
Gender	
Male	13
Female	10
Average age (range)	51 (17–71)
Smoking history	4
Comorbidity	
Diabetes mellitus	4
Obesity (BMI > 28)	2
Hypertension	7
Osteoporosis (T score < − 2.5)	5
Immune system disorder	1*
Site	
Thoracic	3
Thoracolumbar	13
Lumbar	7
Vertebra involved	
Two vertebrae	20
Three vertebrae	3
ASIA grading	
ASIA C	2
ASIA D	5
ASIA E	16
VAS	7.5(5–9)
Average CRP **mg/L (range)**	30.6(15.1–55.8)
Average ESR mm/h (range)	72.3(45–120)

*One patient was diagnosed with vitiligo

### Inclusion criteria

Patients who had one or more of the following characteristics were included in this study: (1) be bed ridden by severe back pain even after 3 weeks ATT, (2) neurological deficit caused by spinal canal involvement, (3) large sequestrum formation or large vertebral body destruction, (4) significant spinal instability (a sagittal plane displacement greater than 2.5 mm in the thoracic spine on lateral radiograph was considered unstable, a sagittal plane displacement greater than 4.5 mm in the lumbar spine, or 15 % of the anteroposterior diameter of the vertebral body on a static lateral radiograph, was considered potentially unstable) [15], (5) the progression of kyphosis angle more than 5° during ATT period, and (6) aged patients could not bear long time bed rest.

### Exclusion criteria

Patients with the following conditions were excluded in this study: (1) kyphosis angle more than 40°, (2) the affected vertebral body collapse greater than 1/2 of its total height, and (3) poor general condition made the procedure impossible.

### Preoperative management

Medical history and physical examination were acquired for each patient after admission; spinal cord function was evaluated using the American Spinal Injury Association (ASIA) grading. Plain x-rays, computerized tomography (CT), and magnetic resonance image (MRI) were used to evaluate spinal pathologies such as osteopenia, paravertebral abscess, disc space destruction, vertebral body collapse, and endplate erosion. Localized kyphotic deformity was evaluated by Cobb’s methods (the angulation of the upper and lower endplates of the collapsed vertebral body). In addition, the following laboratory tests were mandated for each patient at the first visit: complete blood count, erythrocyte sedimentation rate (ESR), C-reaction protein (CRP), tuberculosis-antibody (TB-Ab), and T-SPOT. The diagnoses were based on the clinical findings, laboratory investigation, and imaging features and were confirmed by preoperative or intra-operative biopsy.

The anti-tuberculous chemotherapy (ATT) was applied to each patient for two to three weeks before surgery. The chemotherapeutic regimens include isoniazid (5 mg/kg, 300 mg/day), rifampicin (15 mg/kg, 600 mg/day), pyrazinamide (25 mg/kg/, 2 g/day), and ethambutol (15 mg/kg, 750 mg/day). ESR was monitored during the ATT. Other treatments included improvement in the nutritional status, painkillers, and limitation of lower back activities.

### Surgical procedure

Under general anesthesia, the patient was positioned prone on an Allen spinal surgery bed, and care should be taken to keep the abdomen free-hanging. All the targeted segments were confirmed using fluoroscopy. The skin incisions were marked according to the fixation type we used, which include long segmental fixation and short segmental fixation.

**Fig. 1:**
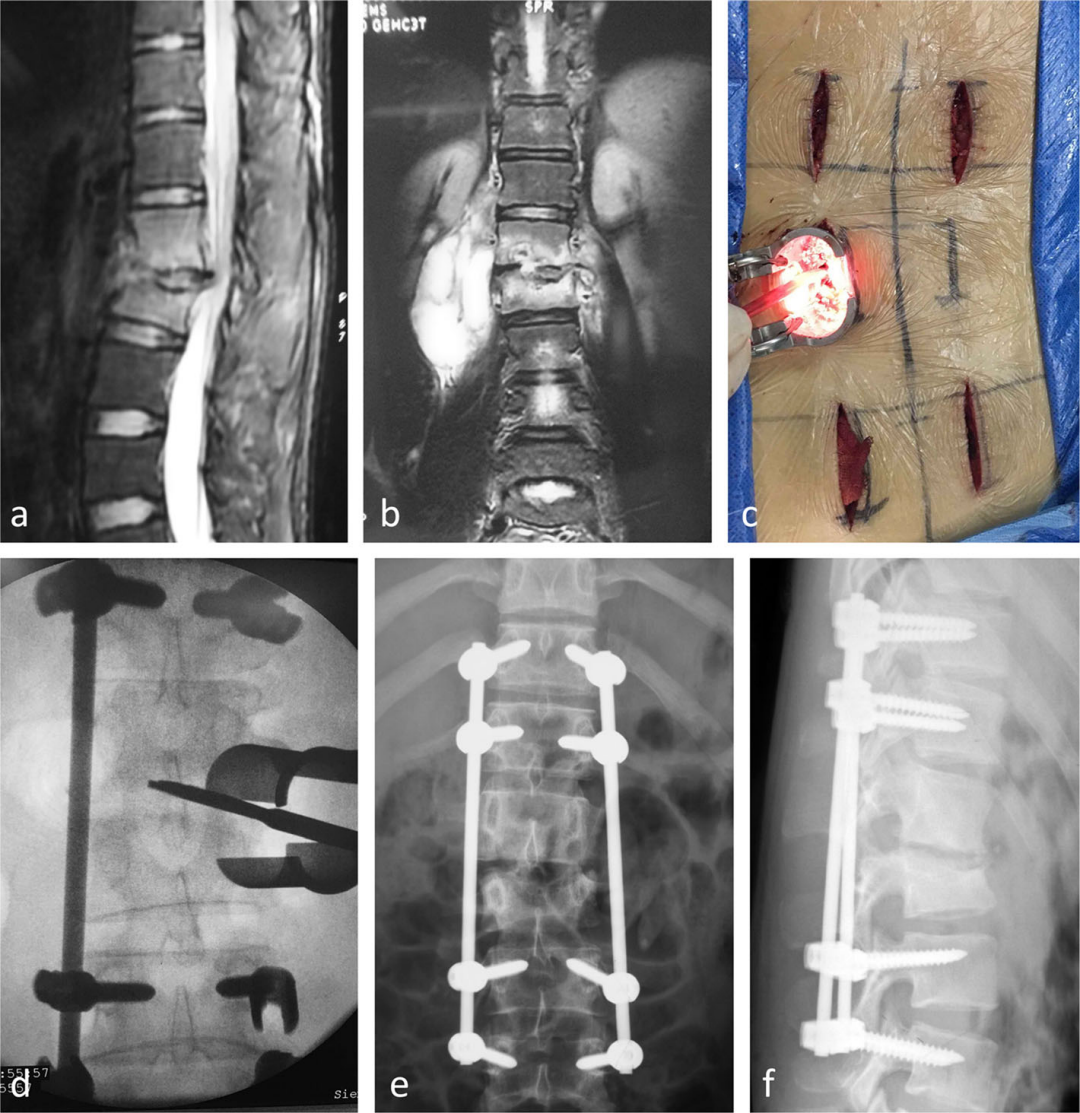
A case of a 16-year-old male suffered from back pain with L1/2 tuberculosis. **a** Preoperative sagittal MRI showed tuberculosis which involves the intervertebral disc, leading to the kyphosis. **b** Preoperative coronal MRI showed psoas abscess and destruction of intervertebral disc. **c** Five 3 cm-long incisions were carried out, four of them for the pedicle screws insertion, and the fifth for debriding the TB lesion. **d** Expandable tubular retractor was docked over the right side facet joint, and the TB lesion was cleared through the far lateral approach. **e**, **f** Postoperative anteroposterior and lateral radiographs after long segmental fixation showed acceptable spinal alignment

In the long segmental fixation group, the pedicle screws were only inserted in the adjacent normal vertebrae. This kind of fixation was used in early 15 cases to eliminate the interference of the instrumentation to the TB focus. In this group, a total of five 3 cm-long longitudinal incisions were needed. Four incisions were made symmetrical and paired 2–3 cm away from the posterior midline. Each incision was able to provide access to adjacent two vertebrae for ipsilateral two pedicle screws insertion. The fifth incision was made on the more severe side of involved vertebra for the debridement of TB lesions (Fig. 1a–f).

Unilateral pedicle screw fixation should be accomplished at first. After the skin was cut open, the deep fascia was incised, paraspinal muscles were split by passing the sequential dilators, and the expandable tubular retractor (Quadrant, Medtronic, USA) was docked over the facet joint. After the insertion of the unilateral four screws, a 6-cm rod was installed and locked with nuts. Following placement of the “stability” rod on the less involved side, the remaining screws were inserted in the vertebrae on the other side. However, the rod should not be installed until the TB lesions were removed through the fifth incision.

**Fig. 2:**
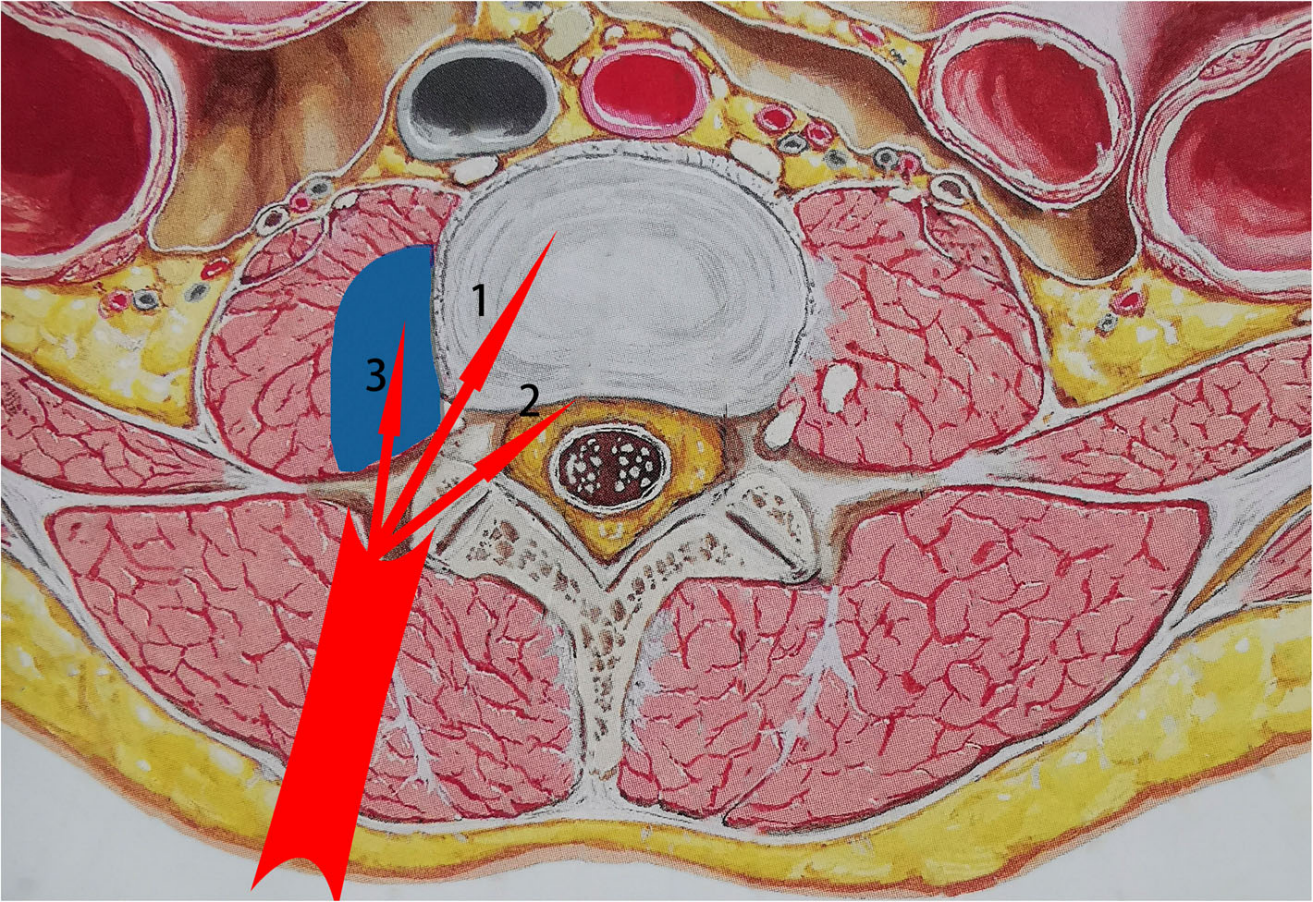
The routes of TB debridement. Arrow 1: go anteromedially into the involved disc along the lateral side of the SAP. Arrow 2: go more medially to the canal by resecting part of the anterolateral side of the SAP. Arrow 3: go anteriorly to the psoas abscess

In the fifth incision, after the retractor was docked, we went deeply along the lateral side of the superior articular process (SAP) to reach the posterolateral side of the involved disc. Care should be taken to dissect and protect the exiting nerve root which is usually cranial to the disc. For cases without spinal canal involvement, we went anteromedially into the disc along the SAP to eradicate the purulent material, the granulation tissue, and the necrotic bone fragments. For cases with spinal canal involvement, we went more medially by resecting part of the anterolateral side of the SAP and the ligamentum flavum. When the shoulder of the transversing nerve root was exposed, the canal decompression could be done by using a nerve hook or a small curved curet. For patients with psoas major abscess, we went anteriorly to the abscess to release the pus before accessing the disc (Fig. 2).

In the short segmental fixation group, at first, four pedicle screws were percutaneously and ipsilaterally inserted into the involved vertebra, and the adjacent two normal vertebrae with a 5.- cm rod assembled to provide intraoperative stability. Secondly, two pedicle screws were inserted into the other side of the adjacent normal vertebrae for the final stability after debridement. The debridement procedure was the same as that in the long segmental group.

After the debridement, a ventricular drainage tube was placed in the residual interbody cavity for later anti-tuberculous drugs administration. Superficial wound drainage was not necessary because of the less bleeding inside the small incision.

### Postoperative management

Patients were encouraged off-bed activities on the second postoperative day with a thoracolumbar brace after 1-day back muscle stretch exercise in bed. One hundred milligrams of isoniazid was injected into the interbody cavity through the ventricular drainage tube from the third postoperative day until the drainage and sutures were removed within 2 weeks. ATT was continued for 9–12 months (isoniazid, rifampicin, ethambutol, and pyrazinamide) according to the lab results and the imageology except for a drug-resistant patient who were treated with second line ATT (levofloxaxin and pasiniazid) for 24 months. Radiographs were taken immediately after surgery at 1, 3, 6, and 12 months postoperatively (24 months in the case with drug resistance) to evaluate the spinal alignment and the validity of the fixation. The brace was discontinued 3 months after surgery. The long segmental instrumentation was recommended to be removed 1.5 years after surgery when the clinical cure was assumed.

### Statistical analysis

All data were presented as means ± standard deviations (*x* ± *s*). The ESR, VAS, CRP, ODI, and Cobb’s angle at pre-surgery, post-surgery, and the last follow-up were compared using paired *T* test. *P* < 0.05 was considered to be statistically significant. All analyses were conducted using the SPSS 19.0 software (SPSS, Inc., Chicago, IL, USA).

## Results

All surgeries were successfully performed by the senior author. The patients were followed for an average of 19 months, ranging from 12 to 36 months. The mean intraoperative blood loss was 223 ± 97 ml, and the mean operative time was 165 ± 42 min. Twenty two patients showed definite and persistent clinical response to ATT, which was confirmed by clinical manifestations and laboratory investigations. Only a young female who presented with severe back pain and rapid progression of the vertebral destruction was still irresponsive to standard four-drug ATT regimen for 3 weeks. She was proved to be suffering from drug-resistant tuberculosis after surgery, and second line anti-tubercular drugs were administrated for 2 years to reach the clinical cure. The criteria for clinical cure include the following: (1) good general condition with normal appetite, no fever, no back pain, (2) consecutive normal ESR, (3) bone healing around the vertebral bodies on CT scan, and no high-intensity around the focus on T2 weighted MRI, and (4) no signs of recurrence after more than 3 month daily activity.

### Clinical outcomes

The CRPs and ESRs were normal at the final follow-up (Table 2). The VAS and ODI were significantly improved compared with preoperative values (*p* < 0.05). All the patients with preoperative neurological deficit showed complete recovery at the last follow-up (Table 3).

**Table 2:** The changes of ESR VAS, CRP, ODI, and Cobb’s angle after surgery

	Pre-op	2 weeks post-op	Last follow-up	*p*1	*p*2
ESR	64.5 ± 24.4	47.3 ± 18.3	13.1 ± 3.7	< 0.05	< 0.05
VAS	7.6 ± 0.9	5 ± 1.1	1.5 ± 0.7	< 0.05	< 0.05
CRP	23.2 ± 11.7	12.9 ± 7.3	4.3 ± 1.7	< 0.05	< 0.05
ODI	74.2 ± 6.7		26.5 ± 6.8		< 0.05
Cobbs	23.2 ± 5.5°	13.2 ± 5.3°	13.4 ± 5.6°	< 0.05	0.33

*p1* the comparison between pre-op and 2-week post-op, *p2* the comparison between 2-week post-op and last follow-up

**Table 3:** Neurological changes after surgery

Pre-op ASIA grading		Last follow-up ASIA grading				
		A	B	C	D	E
C	2					2
D	5					5
E	16					16

### Radiological evaluation

**Fig. 3:**
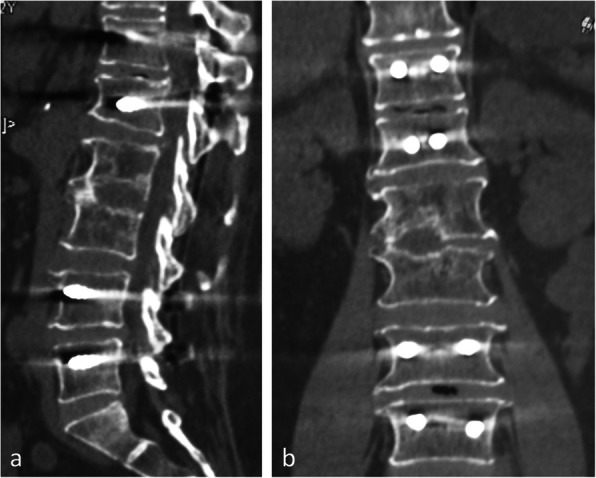
A 62-year-old female, sagittal (**a**) and coronal (**b**) CT reconstruction 1 year after surgery, showed spontaneous peripheral interbody fusion

The Cobb’s angle improved significantly from preoperative 23.2 to 13.2° and maintained at 13.5° at the last follow-up (Table 2); no evident progression of the kyphotic deformity was found during the follow-up period (*P* > 0.05). Twenty-two patients showed spontaneous peripheral interbody fusion 1 year after surgery (Fig. 3a, b).

**Fig. 4:**
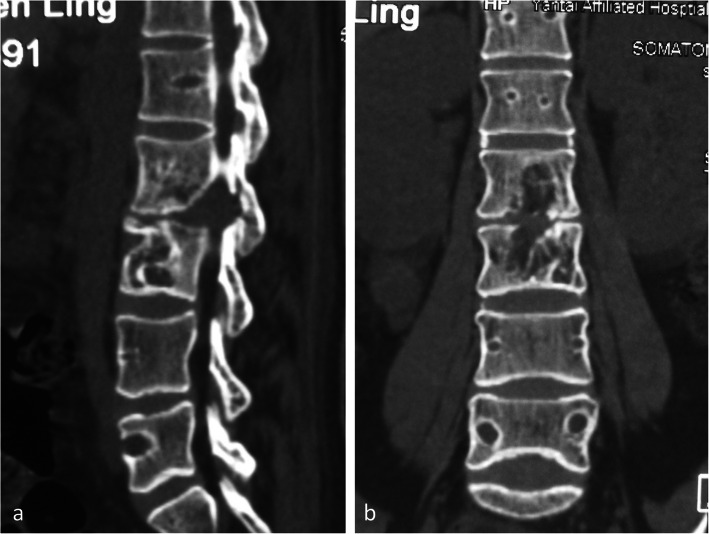
A 28-year-old girl with drug-resistant tuberculosis, sagittal (**a**) and coronal (**b**) CT reconstruction after instrumentation removal at the third year follow-up, showed no signs of intervertebral fusion, but the periphery healing of the vertebrae and preservation of bilateral facet joints provide sufficient support to anterior and posterior column to ensure the spinal stability

**Fig 5.**
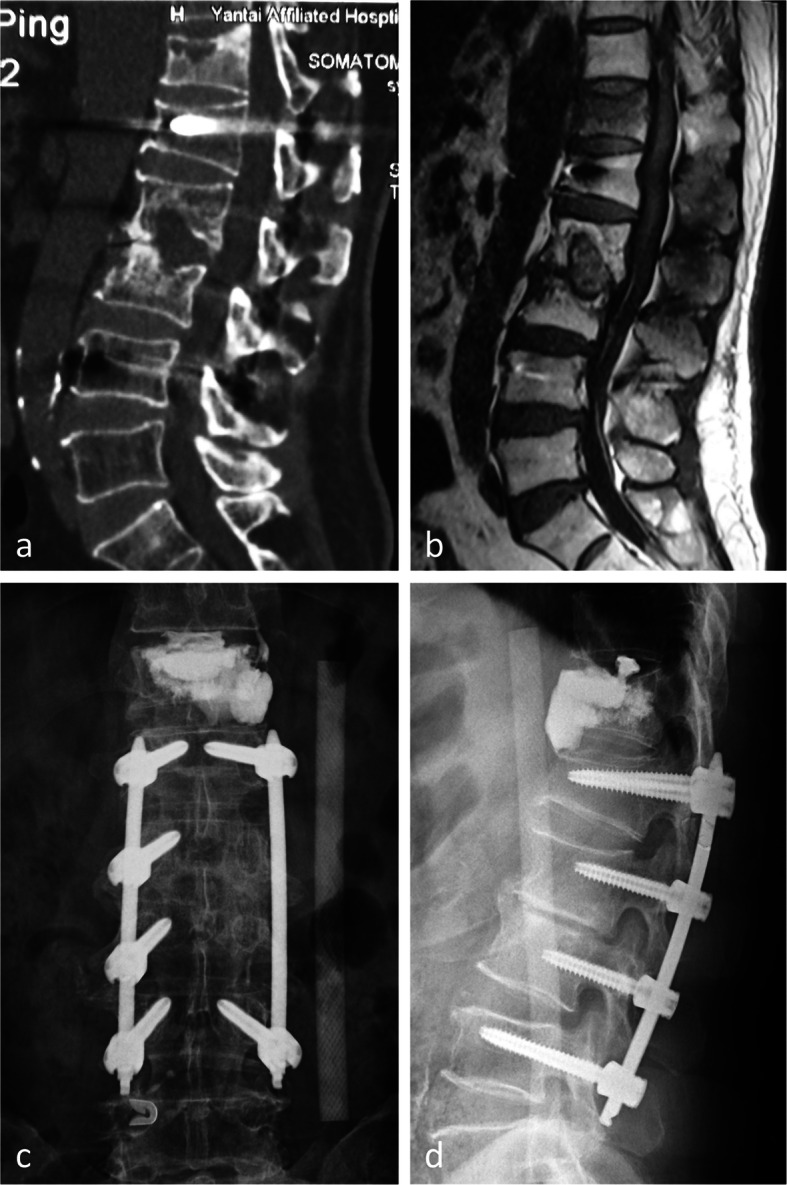
A 60-year-old man suffered from back pain following a fall at 6 months after STB surgery. **a**, **b** The CT and MRI showed adjacent segment compression fracture. **c**, **d** Anteroposterior and lateral radiograph after percutaneous kyphoplasty

There were no failure of the fixation even the case of the girl with drug-resistant tuberculosis that showed no sign of interbody fusion at the third year follow-up (Figs. 4a and 3b). One patient in short segmental fixation group suffered adjacent vertebral body compression fracture resulting from a fall 6 months after his index surgery, and he was able to recover daily activities after a percutaneous kyphoplasty was performed (Fig. 5a–d). No other complications were found in this series of patients.

## Discussion

Tuberculous spondylitis, which is popularly known as Pott’s spine, is the most frequently encountered extrapulmonary form of tuberculosis. Early diagnosis and prompt treatment are essential to avoid neurological disability and minimize spinal deformity [5, 16]. Although anti-tuberculous treatment alone can be effective, surgery should be considered in some circumstances with the benefits of quicker pain relief, earlier ambulation, less kyphosis deformity, immediate neurological decompression, and less relapse. The indications for surgery were suggested to be vertebral lesions, refractory disease, severe kyphosis, progressive neurological deficit, and clinical deterioration or lack of clinical improvement [17, 18]. The surgical indications of this series of patients include (1) bed ridden by severe back pain even after 3 weeks’ ATT, (2) neurological deficit caused by spinal canal occupation, (3) sequestrum formation or large vertebral body destruction, (4) significant spinal instability, (5) progressive kyphosis more than 5° during the ATT period, and (6) aged patient could not bear long time bed rest. Severe kyphosis (more than 30°) cases were not included in this study for the possibility of large bone defect left after surgery.

Many studies have confirmed the efficacy of the surgical treatment of spinal tuberculosis through anterior, anterolateral, posterior debridement and intervertebral fusion or combined with internal fixation to improve the neurological deficit, and correct the kyphotic deformity [19, 20]. However, the conventional surgical approach requires extensive exposure of the affected areas with high rates of morbidity and mortality. Further latent hazards are prolonged surgery, increased blood loss, extensive iatrogenic paraspinal muscle damage, longer hospital stay, and rehabilitation time. Minimally invasive surgery (MIS) via a tubular retractor system was developed for spinal infection [21, 22]. Kandwal et al. [23] reported minimally invasive transforaminal (Mi-TF) approach through a tubular retractor for the treatment of thoracic tuberculosis. However, the damages to the facet joint and the disturbances to the spinal canal in this procedure were theoretically inevitable. Yang et al. [24] described minimally invasive interlaminal approach in the treatment of spine tuberculosis. Through this approach, they did spinal canal decompression and debridement without any fixation or bone grafting. However, the candidates for this procedure were adult patients with mild anterior column destruction due to the concern of progressive kyphosis. Ying et al. [25] described mini-open anterior approach focal clearance combined with posterior internal fixation for lumbar tuberculosis. The anatomical challenge within a small incision and the disturbance to the peritoneum were the limitations of this technique. Yang et al. [26] described local chemotherapy combined with percutaneous pedicle screw in the treatment of spine tuberculosis. The kyphosis was partially corrected, and the progression was prevented by the fixation. However, one of the cases developed serious abscess in the primary focus, and an open surgery was needed. The absence of debridement in this technique might have left too much burden of TB to antituberculous drugs in this case, which finally led to the abscess formation. In our study, the debridement and pedicle screw fixation were performed with Quadrant system to minimize the damage to the paraspinal muscles and other posterior elements. Since the blood supply of the paraspinal muscles were maintained nearly intact, there would be less risk of TB spreading through the muscles to form a sinus. The function of the facet joints was preserved well enough to maintain the sagittal stability of the spine, even after the decompression of the canal was performed. In addition, the less blood loss in this procedure is especially favorable to elderly patients in terms of maintaining intraoperative hemodynamic stability.

The minimally invasive far lateral (MI-FL) approach developed in this study for interbody debridement was different from direct lateral (DL) [12], extreme lateral (XL) [13], and oblique lateral approach (OL). Firstly, the incision of the FL approach is 2–3 cm aside the posterior midline. It goes through the paraspinal muscles, which is different from the abdominal muscles in the DL, XL, and OL approaches. Secondly, the MI-FL approach goes medially into the posterolateral side of the disc along the lateral side of SAP while the DL, XL, and OL approaches go directly to the lateral side of the disc. The advantages of the MI-FL approach include the folllowing: (1) there is no disturbance to the peritoneum, the abdominal vessels, the psoas muscle, and the lumbosacral nerves, and (2) it is easy to go into the spinal canal by removing anterolateral part of the SAP to decompress the canal, which is necessary for patients with neurological deficit. Even after the partial removal of the lateral SAP, the facet joint function was still remained. In summary, the MI-FL approach has the same advantages as DL, XL, and OL in terms of maintaining the function of posterior elements, while it gets around the ventral anatomy challenges.

Local continuous chemotherapy without surgery or after surgery [27] was recommended to increase the concentration of anti-tuberculosis drugs in the focus, inhibits the progression of pathological changes, promotes the limitation of the focus, and healing of the lesion. Some authors believed that inadequate eradication of M. tuberculosis organisms in the lesion was the primary cause of sinus formation and recurrence [28]. It was also found that interbody fusion time was significantly shorter in patients treated with postoperative continuous-irrigation chemotherapy and postural drainage than patients without local chemotherapy [29]. We introduced ventricular drainage tube to the interbody cavity not for continuous drug irrigation but a daily intermittent aspiration and drugs injection to ensure the eradication of the possible remnant M. tuberculosis organisms by the highly concentrated drugs. The majority of the patients recovered quickly not only systemically but locally. We attribute the early peripheral interbody fusion to the efficacious local control of the TB with debridement and anti-tubercular drugs injection. Unfortunately, a patient with drug resistance was not recognized until sinus formation was found 3 weeks after surgery, even with the injection of isoniazid into the cavity for 2 weeks. Another debridement was performed only in the soft tissue to remove the sinus tract, and the wound healing was achieved in the end.

The concern for the kyphosis in the treatment of spinal TB was warranted for it can leave long-term deformity even when the infection could be controlled well finally. After conventional open surgery, there was a significant difference in kyphosis angle especially in the pathological thoracolumbar and lumbar regions, and there was a loss of only 1–3° of the kyphosis angle during long-term follow-up [11]. However, in the chemotherapy alone group, the average angulation was found to have increased significantly from 6.25 to 12.36° at the 2-year follow-up [15]. In another study, the author placed a double lumen tube into the lesion center for the administration of large dose isoniazid. It was surprisingly founded that the kyphotic angle had decreased from preoperative 16.47 to 13.35° at the final follow-up [28]. However, in this study, patients with low back pain were confined to bed till the pain was relieved. The improvement of the kyphosis, from our point of view, was the result of postural reduction during long-term bed rest. In our patients, the Cobb’s angle changed significantly from preoperative 23.2 to 13.2° and kept 13.5° at the last follow-up. We attribute the postoperative improvement of the kyphosis to the intraoperative postural reduction and manual manipulation before the instrumentation. Although the kyphosis was not corrected completely, the final result was acceptable considering the progression of the kyphosis was arrested by peripheral interbody fusion in the long run.

Bone grafting was not performed after interbody debridement in our patients initially on account of the concern for the incomplete clearance of the lesions tending to turn the graft into the culture medium for the bacteria. Since the posterior elements of the spine were reserved nearly intact, we primordially believed that the fibrous tissues formed in the interbody cavity after the clinical cure would provide enough anterior support, like disc tissues, maintaining the long-term spinal stability even after the instrumentation was removed. In addition, we surprisingly found that majority of the patients had achieved peripheral interbody fusion within 1 year after surgery with the exception of a drug-resistant patient. Even in this patient, the peripherally healed vertebral bodies touched each other, providing enough anterior support and making the posterior implants unnecessary 2 years after surgery. As a consequence, we are trying to conclude that the interbody bone grafting after debridement is not a necessity in minimally invasive surgery for the spontaneous interbody fusion and posterior elements preservation.

Several limitations of our study should be pointed out. First, the sample size was comparatively small, involving only twenty-three patients. Although the majority of our patients had achieved interbody fusion without bone grafting, and even in one non-union patient, the peripherally healed vertebral bodies touched each other, providing enough anterior support, and we are not sure whether the excellent clinical result will be persistent in a larger group of patients. Second, we did not know what would actually happen if the bone grafting was performed after minimally invasive debridement and instrumentation. Thus, a randomized controlled study should be performed to understand whether the bone grafting is necessary in these cases with minimal kyposis. Third, two fixation methods were used in this study, both of which played the same role in our surgical strategy; however, it would be more convincing if the fixation methods were uniform. Further study in a larger group is needed to find out the minute differences between the two fixation methods, including adjacent segmental disease, TB recurrence, fixation failure, and union rate.

## Conclusion

MI-FLDPI using expandable tubular retractor could be recommended to treat thoracic and lumbar spine tuberculosis for the advantages of less trauma, earlier recovery, and less complications. Spontaneous peripheral interbody fusion was generally observed in patients treated with MI-FLDPI even without bone grafting, indicating that bone grafting is not a necessity when the posterior elements are well preserved in a minimally invasive procedure.

## Data Availability

The data analyzed during the current study are available from the corresponding author on reasonable request.

## Abbreviations

MI-FLDPI: Minimally invasive far lateral debridement combined with posterior instrumentation
MIS: Minimally invasive surgery
MI-FL: Minimally invasive far lateral (approach)
MI-TF: Minimally invasive transforaminal (approach)
DL: Directi lateral (approach)
XL: Extreme lateral (approach)
OL: Oblique lateral (approach)
STB: Spinal tuberculosis
ASIA: American Spinal Injury Association
SAP: Superior articular process
ATT: Anti-tuberculous therapy
CT: Computed tomography
MRI: Magnetic resonance imaging
ESR: Erythrocyte sedimentation rate
CRP: C-reaction protein
VAS: Visual Analogue Score
ODI: Oswestry Disability Index
